# Relationship between depressive symptoms, burnout, job satisfaction and patient safety culture among workers at a university hospital in the Brazilian Amazon region: cross-sectional study with structural equation modeling

**DOI:** 10.1590/1516-3180.2021.0614.15092021

**Published:** 2022-04-11

**Authors:** Marcélia Célia Couteiro Lopes, Carmen Conceição Carrilho Oliva, Nádia Maria Soares Bezerra, Marcus Tolentino Silva, Tais Freire Galvão

**Affiliations:** I MSc. Pharmacist, Postgraduate Pharmaceutical Sciences Program, Universidade Federal do Amazonas (UFAM), Manaus (AM), Brazil.; II MSc. Pharmacist, Postgraduate Pharmaceutical Sciences Program, Universidade Federal do Amazonas (UFAM), Manaus (AM), Brazil.; III MBA. Health Inspector, Department of Health Surveillance, Municipal Health Department of Manaus, Manaus (AM), Brazil.; IV MSc, PhD. Professor, Postgraduate Pharmaceutical Sciences Program, Universidade de Sorocaba (UNISO), Sorocaba (SP), Brazil.; V MSc, PhD. Professor, School of Pharmaceutical Sciences, Universidade Estadual de Campinas (UNICAMP), Campinas (SP), Brazil.

**Keywords:** Burnout, professional, Depression, Job satisfaction, Patient safety, Health personnel, Brazil, Depressive symptoms, Culture of patient safety, Healthcare workers, Amazon

## Abstract

**BACKGROUND:**

Workplaces can be sources of mental distress. In healthcare services, this can also affect patients.

**OBJECTIVE:**

To assess the prevalence of and factors associated with depressive symptoms, burnout, job satisfaction and patient safety culture and the relationships between these constructs, among healthcare workers.

**DESIGN AND SETTING:**

Cross-sectional study in a university hospital in Manaus, Brazil.

**METHODS:**

Randomly selected workers were interviewed based on Brazilian-validated tools. We calculated the prevalence ratio (PR) and 95% confidence interval (CI) of depressive symptoms and burnout using Poisson regression with robust variance; and the β-coefficient of safety culture and job satisfaction using linear regression. Outcome relationships were assessed using partial least-squares structural equation modeling.

**RESULTS:**

300 professionals were included; 67.3% were women. The prevalence of depressive symptom was 19.0% (95% CI: 14.5; 23.5%) and burnout, 8.7% (95% CI: 5.2; 12.3%). Lack of work stability increased depression (PR = 1.88; 95% CI: 1.17; 3.01) and burnout (PR = 2.17; 95% CI: 1.03; 4.57); and reduced job satisfaction (β = -11.93; 95% CI: -18.79; -5.07). Depressive symptoms and burnout were positively correlated, as also were job satisfaction and safety culture (P < 0.001); job satisfaction was negatively correlated with burnout (P < 0.001) and depression (P = 0.035).

**CONCLUSION:**

Impermanent employment contracts increased depression and burnout and reduced job satisfaction. Job satisfaction reduced poor mental health outcomes and increased safety culture. Job satisfaction and safety culture were directly proportional (one construct increased the other and vice versa), as also were depression and burnout. Better working conditions can provide a virtuous cycle of patient safety and occupational health.

## INTRODUCTION

Negative psychological effects are frequently experienced by healthcare professionals, due to distress relating to patients’ sorrows, health-threatening diseases and poor working environments. Workplace conditions within healthcare affect both the health of the workers and the safety of care.^1^ Adverse events and harm to patients cause physical, psychological and professional distress and require proper organizational support for the team.^2^ Frequent exposure to this insalubrious setting affects the emotional health of workers, who face high prevalence of mental health problems.^3,4^ In this highly human-mediated activity, practitioners’ and patients’ health are mutually influenced.^5^

Chronic workplace stress that is not properly managed has been recognized as a cause of burnout syndrome, which was included as a morbidity in the International Classification of Diseases for Mortality and Morbidity Statistics, eleventh revision, in 2019.^6^ Satisfaction and engagement with labor are central to wellbeing and good performance at both the individual and the collective level.^7^

The relationships between work conditions, workers’ health and patient safety need further investigation. A review on professional burnout, depression, employee commitment, patient outcomes and safety culture interplays identified that even though safety culture and clinical errors were both associated with burnout, few studies had focused on engagement and individual-level factors that impact patient safety culture.^8^ A previous Brazilian survey observed an inverse causal relationship between burnout syndrome and job satisfaction, while depressive symptoms were predictors for burnout.^9^ Real-world evidence about the connections between safety culture, job satisfaction, depression and burnout could enlighten the discussions and improve both workplace and patient safety.

Safety culture was deemed to be fragile, with predominance of individual culpability standards, in a previous survey at a university hospital in the Brazilian Amazon region.^10^ This scenario motivated an investigation on how patient safety culture, workers’ engagement and outcomes interact in this setting.

## OBJECTIVE

The aim of this study was to assess the prevalence of and factors associated with depressive symptoms, burnout, job satisfaction and patient safety culture and the relationships between these constructs, among healthcare workers at a university in the Brazilian Amazon region.

## METHODS

### Study design and setting

This was a cross-sectional study conducted among healthcare workers at Getulio Vargas University Hospital from July to November 2016. This is a teaching hospital belonging to the Universidade Federal do Amazonas (UFAM), and it forms part of the Brazilian National Health System (Sistema Único de Saúde, SUS). At the time of this study, the hospital had 159 beds (11 in intensive care) and 1,222 employees.

### Participants

Individuals who had been employees for at least three months and who were working in the main building of the hospital were eligible for inclusion in this study. The sample size was calculated as 300 participants, considering the potential total population of 863 eligible employees, a frequency of positive answers regarding safety culture of 50%, a confidence level of 95%, design effect of 1 (random sampling) and addition of 10% to compensate for losses. From the hospital’s human resources spreadsheet, we randomly selected 300 employees for interviews and 300 employees to serve as replacements in cases of refusals.

### Variables

The primary outcomes were the prevalences of depressive symptoms and burnout, and the perceptions of job satisfaction and patient safety culture.

The independent variables were the following: sex (women or men); age (in years, categorized as 18-35, 35-50 or 51 or over); skin color/race (whites [white and Asian] or non-whites [black, indigenous and brown, i.e. Brazilian mixed race]); marital status (married or single [single, separated, divorced or widowed]); body composition (normal, overweight or obese); educational level (high school or less, higher education or postgraduate school); economic classification (A, B1, B2 or C/D/E); profession (physicians, nursing staff [nurses and nursing technicians], other healthcare professionals [psychologists, nutrition/radiology/laboratory technicians, physiotherapists, social workers and nutritionists] or technical support [maintenance/cleaning and administrative staff, i.e. office assistant/secretary/receptionist]); working in direct contact with patients (yes or no); length of time working in the hospital (in years, categorized as < 1, 1-2, 3-4, 5-10, 11-20 or ≥ 21); work contract stability (permanent [statutory, contracted through consolidation of labor laws] or temporary [contracted through a support foundation or residency scholarship]); number of jobs (1, 2 or ≥ 3); and weekly workload (in hours, categorized as 20-40, 41-60 or ≥ 61).

### Data sources and measurement

The participants who had been drawn for inclusion were contacted by the research team and were asked to answer a questionnaire using electronic devices (Samsung Tab-3 SM-T110). The KoboToolbox software (https://www.kobotoolbox.org/) was used to configure the questionnaire, with mandatory answers for each question. The software worked offline and filled-out forms were submitted automatically once an internet connection was available.

Depressive symptoms were measured using the nine-item Patient Health Questionnaire (PHQ-9), in its version validated for use in Brazil.^11^ We adopted the cutoff point of ≥ 9 to define presence of depressive symptoms.^12^ The Brazilian version of the Maslach Burnout Inventory Human Services Survey (MBI-HSS) was used to assess burnout, which was taken to be present in cases of higher scores for emotional exhaustion (≥ 27) and depersonalization (≥ 13), and lower scores for personal accomplishment (≤ 31).^13^

Job satisfaction was assessed through the validated version of the Job Satisfaction Survey (JSS) for the Brazilian context.^14^ The score range of 36-108 was taken to indicate dissatisfaction; 144-216, satisfaction; and 109-143, ambivalence. Safety culture was measured through the Brazilian validated version of the Safety Attitudes Questionnaire (SAQ). After reversing the scores of negatively worded items, the positivity percentage was calculated and results of 75% or more were considered to denote the presence of a positive safety culture.^15^

The economic classification was based on reported possession of comfort items and on the education level of the head of the family, which led to a classification from A (richest) to E (poorest).^16^ Weight and height were self-reported by the participants and body mass index (BMI) was obtained by dividing the weight in kilograms (kg) by the square of the height in meters (m^2^). BMI was classified as normal (≤ 24.9 kg/m^2^), overweight (25-29.9 kg/m^2^) or obese (≥ 30 kg/m^2^). Weekly workload and the number of jobs were obtained from the Brazilian National Registry of Healthcare Establishments by searching for the professional’s name.^17^ Information on work contract stability was obtained from the hospital’s human resources spreadsheet.

### Statistical methods

We calculated descriptive statistics for all variables, and obtained frequencies, means, standard deviations and 95% confidence intervals (CI), according to the nature of the data. The instruments’ reliability was calculated by means of Cronbach’s alpha, using a cutoff of ≥ 0.6.

The prevalence ratio (PR) and 95% CI of depressive symptoms and burnout were calculated by means of Poisson regression with robust variance. Factors associated with safety culture and job satisfaction were calculated through linear regression to obtain the coefficient β and 95% CI. We adopted the significance level of P < 0.05 to identify presence of associations between the outcomes and independent variables.

The relationships between depressive symptoms, burnout, job satisfaction and patient safety culture were assessed using partial least-squares structural equation modeling (PLS-SEM). The significance and the relationships between the constructs were established through the path coefficient, expressed as values between -1 and 1. Values close to 0 indicate weak relationships and Pearson’s coefficient of determination was classified as showing a large effect if R^2^ ≥ 26%.^18^ We tested all relationships between constructs and kept significant associations (P < 0.05) in the final PLS-SEM. All analyses were done using the Stata software, version 14.2 (StataCorp, College Station, Texas, United States).

### Ethics

This project was approved by the Research Ethics Committee of the UFAM, through opinion report no. 1,564,841, dated May 30, 2016 (Certificate of Submission for Ethical Appreciation: 44286115.0.0000.5020, on the Brazil Platform); and by the university hospital’s teaching and research management (research monitoring number 03/2016 NP0 25/2015). Participation in the research was voluntary, and only took place after the individual had read and signed an informed consent statement.

## RESULTS

### Participants’ characteristics

**Figure 1.**
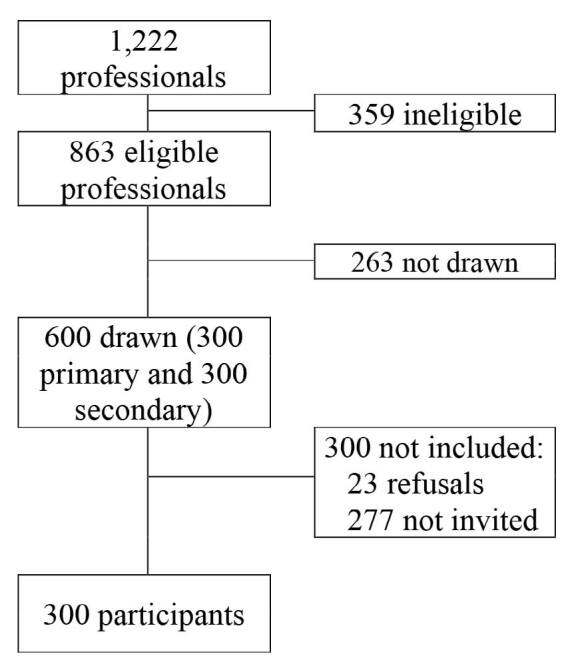
Process of study participant selection and inclusion.

Out of the 1,222 active employees, 863 were eligible and 300 were included. There were 23 refusals among the first 300 invitees, thus yielding initial response rate of 93% (Figure 1).

The majority were women (67%), aged 36-50 years (48%) and married (65%), had nonwhite skin color (black, brown or indigenous; 66%), had a postgraduate degree (53.3%), were overweight (42%) and belonged to economic classification B2 (33%) (Table 1).

**Table 1.** Sociodemographic and professional characteristics of participants and frequencies of depressive symptoms, burnout, job satisfaction and safety culture (n = 300)

Variables	n	%	Depressive symptoms	Burnout	Job satisfaction	Safety culture
			n (%)	n (%)	Mean ± SD	% ± SD
**Sex**						
Women	202	67.3	42 (20.8)	15 (7.4)	131.0 ± 25.6	60.8 ± 13.5
Men	98	32.7	15 (15.3)	11 (11.2)	131.9 ± 25.7	67.3 ± 14.7
**Age (years)**						
18-35	82	27.3	17 (20.7)	12 (14.6)	127.7 ± 23.3	62.0 ± 12.1
36-50	144	48.0	27 (18.8)	9 (6.3)	132.8 ± 27.0	62.8 ± 15.3
≥ 51	74	24.7	13 (17.6)	5 (6.8)	132.5 ± 25.0	63.2 ± 14.1
**Skin color**						
White	103	34.3	21 (20.4)	9 (6.3)	127.4 ± 25.7	60.7 ± 15.6
Nonwhite	197	65.7	36 (13.1)	17 (8.6)	133.4 ± 25.4	63.7 ± 13.1
**Marital status**						
Single	105	35.0	24 (22.9)	15 (8.7)	129.1 ± 28.5	61.8 ± 13.1
Married	195	65.0	33 (16.9)	11 (8.6)	132.5 ± 23.9	63.1 ± 14.7
**Body composition**						
Normal	101	33.7	20 (19.8)	11 (10.9)	129.9 ± 23.6	61.8 ± 11.9
Overweight	126	42.0	25 (19.8)	12 (9.5)	131.8 ± 26.1	63.8 ± 15.1
Obese	73	24.3	12 (16.4)	3 (4.1)	132.5 ± 27.5	62.0 ± 15.2
**Economic class**						
A	77	25.7	17 (22.1)	14 (18.2)	122.9 ± 26.0	62.5 ± 15.6
B1	69	23.0	15 (21.7)	6 (8.7)	130.9 ± 21.7	61.5 ± 13.5
B2	100	33.3	17 (17.0)	5 (5.0)	135.6 ± 25.6	62.9 ± 13.5
C/D/E	54	18.0	8 (14.8)	1 (1.9)	136.1 ± 27.1	64.4 ± 13.8
**Profession**						
Physician	73	24.3	14 (19.2)	11 (15.1)	119.7 ± 22.8	62.8 ± 14.4
Nursing staff	123	41.0	21 (17.1)	5 (4.1)	138.4 ± 24.6	63.4 ± 12.8
Other healthcare professional	57	19.0	11 (19.3)	6 (10.5)	127.5 ± 27.5	61.9 ± 16.2
Technical support	47	15.7	11 (23.4)	4 (8.5)	135.5 ± 23.0	60.4 ± 15.3
**Contact with patient**						
No	252	84.0	9 (18.8)	2 (4.2)	132.3 ± 26.8	56.5 ± 16.7
Yes	48	16.0	48 (19.1)	24 (9.5)	131.1 ± 25.4	63.5 ± 13.6
**Time working in the hospital (years)**						
< 1	13	4.3	4 (30.8)	1 (7.7)	134.5 ± 21.7	62.8 ± 12.4
1-2	28	9.3	7 (25.0)	3 (10.7)	130.2 ± 26.2	62.3 ± 15.0
3-4	32	10.7	8 (25.0)	7 (21.9)	125.9 ± 18.5	61.8 ± 11.0
5-10	56	18.7	9 (16.1)	5 (8.9)	136.6 ± 25.1	65.6 ± 12.5
11-20	88	29.3	15 (17.1)	4 (4.6)	130.0 ± 25.3	63.7 ± 15.0
≥ 21	83	27.7	14 (16.9)	6 (7.2)	131.2 ± 28.8	60.0 ± 15.4
**Contract stability**						
Permanent	233	77.7	37 (15.9)	16 (6.9)	134.0 ± 26.2	62.9 ± 14.4
Temporary	67	22.3	20 (29.9)	10 (14.9)	122.1 ± 20.9	62.0 ± 13.1
**Education**						
Postgraduate degree	160	53.3	24 (15.0)	11 (6.9)	130.3 ± 26.7	61.5 ± 14.8
University/college	73	24.3	24 (32.9)	13 (17.8)	128.2 ± 25.6	63.0 ± 13.3
High school or less	67	22.3	9 (13.4)	2 (3.0)	137.1 ± 22.0	65.4 ± 13.1
**Number of jobs**						
1	172	57.3	43 (25.0)	18 (10.5)	129.8 ± 24.2	60.8 ± 13.6
2	82	27.3	8 (9.7)	6 (7.3)	136.4 ± 28.6	63.9 ± 16.2
≥ 3	46	15.3	6 (13.0)	2 (4.4)	128.3 ± 24.0	66.6 ± 11.9
**Weekly workload (hours)**						
20-40	143	47.7	32 (22.4)	12 (8.4)	132.4 ± 23.7	60.9 ± 14.1
41-60	101	33.7	20 (19.8)	11 (10.9)	128.6 ± 27.5	62.3 ± 15.2
≥ 61	56	18.7	5 (8.9)	3 (5.4)	133.4 ± 26.7	67.4 ± 11.1

SD = standard deviation.

### Frequencies of depressive symptoms, burnout, job satisfaction and safety culture

Depressive symptoms were present in 19.0% (95% CI: 14.5; 23.5%; α = 0.845). The highest prevalences in our sample were noted among people who had been working in the hospital for fewer than five years and among those with higher education, one job and weekly workload of up to 40 hours. Burnout affected 8.7% of the participants (95% CI: 5.2; 12.3%; α = 0.908). Higher prevalence was noted among the participants in higher social classes, physicians, people who had been working in the hospital for 3-4 years and those with higher educational attainment (Table 1).

Job satisfaction scores were ambivalent in the whole sample (131.3; 95% CI: 128.4; 134.2; α = 0.884). Higher scores were observed among nursing staff, people who had been working in the hospital for 5-10 years, those with high school education or less and those with two job contracts. The level of safety culture did not reach the criterion for a positive safety culture that had been defined (i.e. 75%) (62.7%; 95% CI: 60.7; 64.6%; α = 0.920). Higher scores for safety culture were observed among men and professionals with weekly workloads of over 60 hours (Table 1).

### Factors associated with outcomes

Workers with temporary contracts (PR = 1.88; 95% CI: 1.17; 3.01) and with higher education (PR = 2.19; 95% CI = 1.33; 3.59) had higher prevalence of depressive symptoms than workers with stable contracts (Table 2). Those with two jobs (PR = 0.39; 95% CI: 0.19; 0.79) and with weekly workloads of 61 hours or more had fewer depressive symptoms (PR = 0.40; 95% CI: 0.16; 0.97). The prevalence of burnout was lower among people aged 36 to 50 years (PR = 0.43; 95% CI: 0.19; 0.97), married people (PR = 0.39; 95% CI: 0.19; 0.83), individuals in lower economic strata (PR = 0.10; 95% CI: 0.01; 0.75) and nursing staff (PR = 0.27; 95% CI: 0.10; 0.75), in comparison with the respective reference. It was significantly higher among workers with temporary contracts (PR = 2.17; 95% CI: 1.03; 4.57) and with higher education (PR = 2.59; 95% CI: 1.22; 5.51).

**Table 2.** Factors associated with depressive symptoms and burnout, calculated by means of Poisson regression with robust variance; and with patient safety culture and job satisfaction, calculated by means of logistic regression

Variables	Depressive symptoms	Burnout	Job satisfaction	Safety culture
	PR (95% CI)	PR (95% CI)	β (95% CI)	β (95% CI)
**Sex**				
Women	Reference	Reference	Reference	Reference
Men	0.74 (0.42; 1.26)	1.51 (0.72; 3.17)	0.88 (-5.33; 7.09)	6.47 (2.27; 10.68)
**Age (years)**				
18-35	Reference	Reference	Reference	Reference
36-50	0.90 (0.52; 1.55)	0.43 (0.19; 0.97)	5.15 (-1.82; 12.11)	0.86 (-3.69; 5.40)
≥ 51	0.85 (0.44; 1.62)	0.46 (0.17; 1.25)	4.85 (-3.21; 12.92)	1.18 (-4.28; 6.63)
**Skin color**				
White	Reference	Reference	Reference	Reference
Nonwhite	0.90 (0.55; 1.45)	0.99 (0.46; 2.14)	6.03 (-0.07; 12.12)	3.01 (-1.03; 7.05)
**Marital status**				
Single	Reference	Reference	Reference	Reference
Married	0.74 (0.46; 1.18)	0.39 (0.19; 0.83)	3.49 (-2.60; 9.58)	1.30 (-2.74; 5.35)
**Body composition**				
Normal	Reference	Reference	Reference	Reference
Overweight	1.00 (0.60; 1.70)	0.87 (0.40; 1.90)	1.88 (-4.86; 8.62)	2.00 (-2.52; 6.51)
Obese	0.83 (0.43; 1.59)	0.38 (0.11; 1.31)	2.57 (-5.19; 10.32)	0.23 (-4.90; 5.35)
**Economic class**				
A	Reference	Reference	Reference	Reference
B1	0.98 (0.53; 1.82)	0.48 (0.19; 1.18)	7.96 (-0.24; 16.17)	-1.04 (-6.38; 4.3)
B2	0.77 (0.42; 1.40)	0.28 (0.10; 0.73)	12.64 (5.14; 20.15)	0.34 (-4.74; 5.43)
C/D/E	0.67 (0.31; 1.44)	0.10 (0.01; 0.75)	13.18 (4.40; 21.97)	1.92 (-4.17; 8.02)
**Profession**				
Physician	Reference	Reference	Reference	Reference
Nursing staff	0.90 (0.48; 1.64)	0.27 (0.10; 0.75)	18.75 (11.62; 25.88)	0.64 (-4.04; 5.32)
Other health professional	1.01 (0.49; 2.04)	0.70 (0.27; 1.78)	7.87 (-0.66; 16.40)	-0.88 (-6.95; 5.20)
Technical support	1.22 (0.60; 2.45)	0.56 (0.19; 1.67)	15.87 (6.85; 24.90)	2.35 (-9.12; 4.41)
**Contact with patient**				
No	Reference	Reference	Reference	Reference
Yes	1.01 (0.53;1.93)	2.29 (0.56;9.38)	-1.20 (-9.14;6.74)	7.03 (1.05; 13.00)
**Time working in the hospital (years)**				
< 1	Reference	Reference	Reference	Reference
1-2	0.81 (0.28; 2.29)	1.39 (0.16; 12.19)	-4.28 (-21.20; 12.64)	-0.49 (-12.07; 11.08)
3-4	0.81 (0.29; 2.24)	2.84 (0.39; 20.95)	-8.59 (-25.17; 7.99)	-0.98 (-12.29; 10.34)
5-10	0.52 (0.18; 1.43)	1.16 (0.15; 9.14)	2.15 (-13.37; 17.67)	2.82 (-7.90; 13.55
11-20	0.55 (0.21; 1.41)	0.59 (0.07; 4.90)	-4.50 (-19.47; 10.48)	0.88 (-9.70; 11.46)
≥ 21	0.55 (0.21; 1.41)	0.94 (0.12; 7.21)	-3.26 (-18.29; 11.78)	-2.85 (-13.36; 7.67)
**Contract stability**				
Permanent	Reference	Reference	Reference	Reference
Temporary	1.88 (1.17; 3.01)	2.17 (1.03; 4.57)	-11.93 (-18.79; -5.07)	-0.84 (-5.40; 3.72)
**Education**				
Postgraduate degree	Reference	Reference	Reference	Reference
University/college	2.19 (1.33; 3.59)	2.59 (1.22; 5.51)	-2.07 (-9.15; 5.01)	1.40 (-3.24; 6.04)
High school or less	0.90 (0.43; 1.83)	0.43 (0.10; 1.73)	6.77 (-0.52; 14.06)	3.83 (-1.21; 8.86)
**Number of jobs**				
1	Reference	Reference	Reference	Reference
2	0.39 (0.19; 0.79)	0.70 (0.29; 1.70)	6.60 (-0.13; 13.33)	3.04 (-1.62; 7.70)
≥ 3	0.52 (0.23; 1.15)	0.42 (0.10; 1.73)	-1.48 (-9.81; 6.83)	5.80 (0.70; 10.89)
**Weekly workload (hours)**				
20-40	Reference	Reference	Reference	Reference
41-60	0.88 (0.54; 1.46)	1.30 (0.60; 2.83)	-3.83 (-10.38; 2.71)	1.45 (-2.85; 5.75)
≥ 61	0.40 (0.16; 0.97)	0.64 (0.17; 2.18)	1.01 (-6.92; 8.96)	6.57 (1.36; 11.77)

β = beta coefficient; PR = prevalence ratio; CI = confidence interval.

Workers within the fields of nursing (β = 18.7; 95% CI: 11.62; 25.88) and technical support (β = 15.9; 95% CI: 6.85; 24.90) had greater job satisfaction than did physicians. People in lower economic strata had higher job satisfaction than did those in higher strata (P < 0.003); and those with temporary contracts had lower satisfaction than permanent workers (β = -11.93; 95% CI: -18.79; -5.07). Men (β = 6.47; 95% CI: 2.27; 10.68), professionals in direct contact with patients (β = 7.03; 95% CI: 1.05; 13.00), those with three or more jobs (β = 5.80; 0.70; 10.89) and those with weekly workloads exceeding 60 hours (β = 6.57; 95% CI: 1.36; 11.77) had significantly higher perception of safety culture, compared with their reference categories.

### Relationships between depressive symptoms, burnout, job satisfaction and safety culture

**Figure 2.**
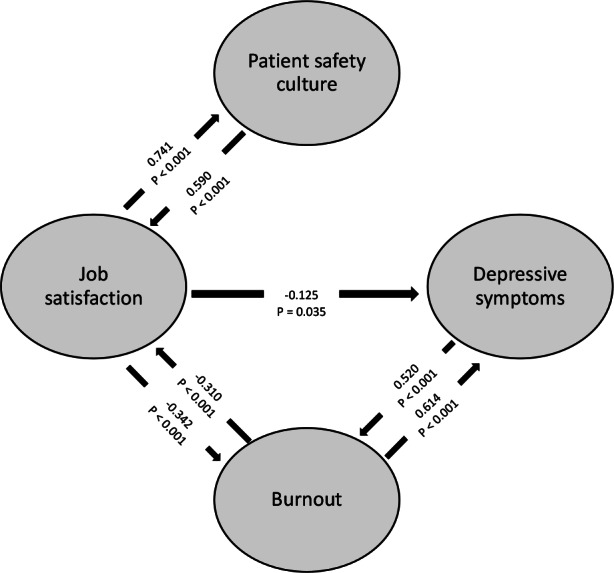
Correlation path of depressive symptoms, burnout, job satisfaction and safety culture, obtained through partial least-squares structural equation modeling (PLS-SEM).

Burnout was negatively correlated with job satisfaction (path coefficient = -0.342; P < 0.001) and it was positively correlated with depressive symptoms (path coefficient = 0.520; P < 0.001); 56% of burnout variance was explained by these factors (Table 3 and Figure 2). Job satisfaction reduced depressive symptoms (path coefficient = -0.125; P = 0.035) and burnout increased these symptoms (path coefficient = 0.614; P < 0.001); 48% of the variability of the depressive symptoms was explained by the predictive variables.

**Table 3.** Correlation between depressive symptoms, burnout, job satisfaction and safety culture

Predictive variable	Endogenous variable	Path coefficient	P-value	R^2^ (%)
Depressive symptoms	Job satisfaction	-0.125	0.035	48.0
	Burnout	0.614	< 0.001	
Burnout	Job satisfaction	-0.342	< 0.001	56.0
	Depressive symptoms	0.520	< 0.001	
Job satisfaction	Safety culture	0.590	< 0.001	62.0
	Burnout	-0.310	< 0.001	
Safety culture	Job satisfaction	0.741	< 0.001	54.8

Job satisfaction increased patient safety culture (path coefficient = 0.741; P < 0.001) and explained 55% of its variance. Safety culture increased job satisfaction (path coefficient = 0.590; P < 0.001) and burnout reduced it (path coefficient = -0.310; P < 0.001); 62% of job satisfaction variance was explained by these predictors. Other path coefficients between the constructs were not significant (P < 0.05) and were removed from the PLS-SEM.

## DISCUSSION

The healthcare workers in this university hospital in the Amazon region had high prevalences of depressive symptoms and burnout, while patient safety culture and job satisfaction were sub-optimal. Burnout and depressive symptoms were directly proportional: the presence of one outcome potentiated the occurrence of the other. Job satisfaction was a protective factor for depression and burnout and was negatively affected by these constructs. Satisfaction with work increased patient safety culture, which in turn improved job satisfaction; and it reduced the frequency of poor mental health outcomes.

Although we used a causality technique to assess how the constructs were related to each other (structural equation modeling), the cross-sectional design did not allow us to draw conclusions regarding causal relationships. No adjustments for confounding factors in regression analyses were performed in order to investigate individual associations: instead, we aimed to study the relationships between constructs.

The present results may have been affected by survival bias, since individuals with severe emotional problems could have been on sick leave or could have taken early retirement, which would thus have rendered them ineligible for this study. Nonetheless, the random sampling adopted and the consequent high response rate increased the level of confidence in the representativeness of this study.

The conclusions from the present study were supported only through quantitative data. Comprehension of the constructs would become better explained if these were supplemented with qualitative data. Measurements were based on a self-administered questionnaire that asked sensitive questions about the workplace and the participants’ mental health of participant. The nature of these questions might have led to non-response bias, but the high reliability that was found for the constructs showed that this risk was low. One contextual factor that could not be avoided was that at the time of data collection, there was co-occurrence of physical renovation and managerial changes, which may have impacted the professionals’ perceptions.

Safety culture and job satisfaction had a positive and reciprocal relationship. Job satisfaction reduced depressive symptoms and burnout and the latter reduced satisfaction. Depressive symptoms and burnout were positively and mutually associated. In a previous survey conducted in a hospital in the state of São Paulo, Brazil, similar relationships between job satisfaction, depressive symptoms and burnout were observed.^9^ An adequate labor environment favors better mental outcomes and safety culture, thereby contributing to the quality of the care provided.

Almost one-fifth of the workers presented depressive symptoms and one-tenth, burnout. The prevalence of depressive symptom was almost three times greater than the 7% observed in the adult population of Manaus Metropolitan Region in 2015,^19^ while the burnout rates were similar to global trends among nurses (10%) and ranged from 9 to 60% among physicians, using the same instruments and cutoffs that we adopted.^20, 21^ Poor mental health outcomes seem to occur more frequently among healthcare workers, and this represents a risk factor for unsafe care.^3-5^ Lack of stability in work contracts doubled the prevalence of both depression and burnout, and significantly reduced job satisfaction in our sample. Concerns about job loss frequently lead to emotional distress.^22^ This situation will probably deteriorate in Brazil with a major labor reform that has been implemented in Brazil, which has increased the occurrence of precarious contracts and has reduced workers’ rights.^23^ Higher pressures on public healthcare, and on healthcare personnel, are also expected because of a freeze on government investments in healthcare and other social areas for 20 years that was started in 2017.^24^

People with two work contracts and with higher weekly workloads had fewer depressive symptoms. This was inconsistent with previous research, in which higher rates of depression due to reduced time for eating, leisure, rest, sleep and social and family contact were observed.^25,26^ The possible explanations for our findings are that coping skills may have been developed through more job experience; or that it may have been a limitations of our cross-sectional design due to reverse causality, i.e. people who fell ill reduced their workload; or that it may have been related to survival bias, as mentioned earlier. Higher workload was also associated with better perception of safety in our sample and in some previous studies,^27,28^ but this was seen to have a negative effect in other settings.^29,30^ The divergences regarding this finding may reflect the labor market and safety culture perceptions of each geographical area,^31^ or possible desensitization to safety issues in situations of longer working hours.

The score for patient safety culture was only slightly above the midpoint and was therefore not considered to be positive culture. The 2015 survey conducted in this hospital also found weaknesses in safety culture, using another instrument.^10^ Men, workers in direct contact with patients and people with greater workloads and more jobs had better perceptions of safety culture. Men also had more favorable perceptions regarding teamwork climate, job satisfaction, management and working conditions in a survey held in the United States using SAQ.^32^ Men tend to present positive reactivity and, according to the model of patterns of change, need more time to perceive potential errors. Thus, they may classify performance as excellent, while this might be considered substandard by women.^33^ A survey among 1,408 professionals also observed higher safety culture among professionals who work in direct contact with patients than among those without contact.^28^ This may reflect the skills developed in adjusting professional and technical issues to achieve a capacity to interrelate with patients.^34^

Job satisfaction was ambivalent in our sample as a whole. Professionals of lower social class and in the field of nursing had higher job satisfaction and lower prevalence of burnout. Job satisfaction probably acted as a protective factor against burnout in these categories. Higher satisfaction in lower social classes is counterintuitive, in that higher income is generally associated with greater satisfaction with payment.^35^ One possible explanation for this is that people of lower economic level place greater value on the type of work performed in hospitals, while those with higher incomes feel disregarded in relation to their working conditions. It is also possible that wealthier people occupy positions that bring more responsibilities, dissatisfaction with work and professional burnout. Lower levels of satisfaction and high prevalence of burnout among both nurses and physicians is a common finding, but comparisons of satisfaction across healthcare professions in the same sample are scarce.^36^ Local reasons can also explain these findings.

More than half of the professionals presented overweight or obesity, without any association with patient safety, job satisfaction, depressive symptoms or burnout. A previous investigation found positive associations between eating problems and burnout,^37^ between burnout and obesity,^38^ and between depression and obesity.^39^ Most of our sample were black, brown or indigenous, and ethnicity was not associated with the constructs in our study, which was a pattern differing from what had previously been observed.^40^

## CONCLUSION

Job satisfaction increased safety culture, which also positively affected satisfaction, and reduced depressive symptoms and professional burnout. Depressive symptoms, burnout, job satisfaction and safety culture were partially explained by and individually associated with lack of work stability, number of jobs, weekly workload and sociodemographic factors. Improving the institution’s patient safety culture involves enhancement of working conditions and empowerment of workers, in order to provide a healthy environment for professionals and safe care for patients. Job satisfaction has a central role in relation to safer care for patients and better mental health among workers.
